# Predictive Factors of Response to Streptozotocin in Neuroendocrine Pancreatic Neoplasms

**DOI:** 10.3390/jcm12247557

**Published:** 2023-12-07

**Authors:** Giuseppe Fanciulli, Anna La Salvia, Sergio Di Molfetta, Giuseppe Cannavale, Giulia Puliani, Monica Verrico, Federica Campolo, Annamaria Colao, Antongiulio Faggiano, NIKE Group

**Affiliations:** 1 Endocrine Oncology Program, Endocrine Unit, Department of Medical, Surgical and Experimental Sciences, University of Sassari, University Hospital of Sassari, 07100 Sassari, Italy; 2 National Center for Drug Research and Evaluation, National Institute of Health (ISS), 00161 Rome, Italy; anna.lasalvia@iss.it; 3 Section of Internal Medicine, Endocrinology, Andrology and Metabolic Diseases, Department of Precision and Regenerative Medicine and Ionian Area, University of Bari Aldo Moro, 70124 Bari, Italy; sergio.dimolfetta@uniba.it; 4 Unit of Endocrinology, Andrology, Diabetology and Nutrition, Department of Clinical Medicine and Surgery, Federico II University, 80131 Naples, Italy; giuseppecannavale95@gmail.com; 5 Oncological Endocrinology Unit, Regina Elena National Cancer Institute IRCCS, 00144 Rome, Italy; giulia.puliani@ifo.it; 6 Department of Radiology, Oncology and Pathology, Sapienza University of Rome, 00161 Rome, Italy; monica.verrico@uniroma1.it; 7 Department of Experimental Medicine, Sapienza University of Rome, 00161 Rome, Italy; federica.campolo@uniroma1.it; 8 UNESCO Chair, Education for Health and Sustainable Development, Federico II University, 80131 Naples, Italy; colao@unina.it; 9 Endocrinology Unit, Department of Clinical and Molecular Medicine, The European Neuroendocrine Tumor Society (ENETS) Center of Excellence, Sant’Andrea Hospital, Sapienza University of Rome, 00189 Rome, Italy; antongiulio.faggiano@uniroma1.it

**Keywords:** neuroendocrine pancreatic neoplasm, streptozotocin, predictors of response

## Abstract

Pancreatic neuroendocrine neoplasms (Pan-NENs) may exhibit a heterogeneous clinical course, ranging from indolent to progressive/metastatic behavior. In the latter scenario, streptozocin (STZ) is considered the cornerstone of systemic treatment; however, response to STZ-based chemotherapy may vary among individuals. In this narrative review, we aimed to identify the predictive factors of response to STZ in advanced Pan-NENs. We performed an extensive search in international online databases for published studies and ongoing clinical trials evaluating STZ in Pan-NENs. We found 11 pertinent studies evaluating 17 patient-, tumor-, or treatment-related factors. Age, CgA blood levels, tumor grade, Ki-67% index, anatomical location of the primary tumor, tumor stage, site of metastasis origin, liver tumor burden, extrahepatic spread, functional status, O6-methylguanine-methyltransferase (MGMT) status, line of therapy, and response to previous treatments were all statistically associated with radiological response and/or survival. The identified predictors may help clinicians make appropriate treatment decisions, in this way improving clinical outcomes in patients with advanced Pan-NENs.

## 1. Introduction

Pancreatic neuroendocrine neoplasms (Pan-NENs) are a heterogeneous group of neoplasms that arise from the neuroendocrine cells of the pancreas. These tumors can exhibit diverse clinical behaviors, ranging from indolent, slow-growing tumors to aggressive and rapidly progressive malignancies [1,2,3]. The classification of NENs has changed over time. The WHO 2022 Classification of Endocrine and Neuroendocrine Tumors divides NENs into well-differentiated neuroendocrine tumors (NETs) and poorly differentiated neuroendocrine carcinomas (NECs). NETs are graded as G1, G2, and G3 based on increased Ki-67 index index, whereas NECs are, by definition, high grade [4]. In addition, Pan-NENs can be classified as functioning or non-functioning tumors, depending on their hormone-secreting activity.

Surgical resection of localized disease is the mainstay of therapy [5], whereas in the setting of advanced disease, systemic treatment is the standard of care. For well-differentiated NETs, the therapeutic armamentarium has progressively increased in recent decades, and comprises biotherapy (somatostatin analogues, SSAs), targeted agents (such as everolimus and sunitinib), interferon, chemotherapy, and radiopharmaceuticals (peptide receptor radionuclide therapy, PRRT). For aggressive, poorly differentiated, metastatic NECs, cytotoxic chemotherapy is the only widely available treatment. In the context of Pan-NENs, streptozocin (STZ)-based chemotherapy is considered an important option in the systemic treatment [6].

STZ (2-deoxy-2-({[methyl(nitroso)amino]carbonyl}amino)-β-D-glucopyranose) is an antibiotic and anticancer drug that was isolated for the first time from *Streptomyces achromogenes* in 1960. STZ has well-known diabetogenic effects due to the selective destruction of pancreatic islet β-cells, and for this reason, has been largely used to induce diabetes in experimental animals [7,8]. Moreover, STZ is an alkylating agent with an established efficacy in Pan-NENs that has led to the approval of this compound on the basis of historical randomized trials [9,10].

STZ has demonstrated its activity as an anticancer drug in Pan-NENs when administered as monotherapy [11], and in combination with other chemotherapeutic agents, including 5-fluorouracil (5-FU) [12] and doxorubicin (DOX) [13]. The indication for STZ use in Pan-NENs therapy varies according to the different international guidelines for Pan-NENs, including the European ones, namely, the European Neuroendocrine tumor Society (ENETS) and the European Society for Medical Oncology (ESMO) guidelines, the National Comprehensive Cancer Network (NCCN), and the Japanese Neuroendocrine Tumor Society (JNETS) guidelines. ENETS, ESMO, and JNETS guidelines indicate STZ use for metastatic pancreatic NETs (Pan-NETs) G1, G2, and G3 but not for pancreatic NECs (Pan-NECs) [14,15,16,17]. Otherwise, according to NCCN guidelines, the level of recommendation for STZ as a therapeutic option for Pan-NENs is lower than the European guidelines [18]. These recommendations are summarized in the Supplementary Table S1.

Moreover, given the lack of head-to-head comparative studies between the above-mentioned therapies, the position of STZ in the treatment algorithm is mainly based on safety/toxicity profiles and comorbidities. The identification of predictive factors of response would then be crucial in the appropriate treatment choice of Pan-NENs. In this context, this narrative review aims to critically evaluate the available data on the potentially relevant predictive factors of response to STZ in Pan-NENs.

## 2. Materials and Methods

We performed an extensive search for published clinical studies employing STZ in Pan-NENs in international online databases (PubMed, Web of Science, and Scopus) using the following terms: neuroendocrine pancreatic tumor, neuroendocrine pancreatic neoplasm, streptozotocin. We included all the studies evaluating STZ (alone or in combination) with a robust statistical methodology (e.g., studies providing the statistical significance and the *p*-value to sustain the results achieved) and excluded data originating from the cumulative analysis of both Pan-NENs and NENs of other anatomic sites, regarding it as not informative/potentially misleading. From 1980 to 2022, different editions of the WHO classification of Pan-NENs have been redacted, containing differences in the nomenclature and grading of tumors. We chose to maintain the terminology provided in each of the selected studies. A schematic overview of the main changes in the subsequent editions of WHO classification of Pan-NENs is provided in the Supplementary Table S2.

With the same keywords used to retrieve published articles, we conducted a search about possible ongoing registered clinical trials (RCTs) on the registries of the US National Institutes of Health, ClinicalTrials.gov, and the European Medicine Agency, Eudract.

The search was last updated on 1 September 2023.

## 3. Results

We detected 11 pertinent published clinical studies. The results are summarized in Table 1. They are listed in the following order:

Patient-related features
  Age
  Performance Status
  Blood biomarkers
  Associated genetic syndromes
Tumor-related features
  Tumor grade
  Ki 67 index
  Anatomic primary tumor site
  Primary tumor size
  Tumor stage
  Site of metastasis origin
  Liver tumor burden
  Extrahepatic spread
  Functional status
  Somatostatin receptors expression
  Mechanisms of DNA repair
Treatment-related factors
  Line of therapy
  Response to prior treatment

As for RCTs, ClinicalTrials.gov and Eudract did not report any trial having as its main or secondary objective the identification of predictive factors of response to SZT in Pan-NENs.

### 3.1. Patient-Related Features 

#### 3.1.1. Age

We found three studies in which age was evaluated as a predictive factor. A recent retrospective analysis was performed on 243 well-differentiated advanced Pan-NETs, selected from a database in the timeframe 1992–2013, who received 5-FU, DOX, and STZ combined chemotherapy regimen (FAS) [19]. The study aimed to assess the objective response rate (ORR) according to the Response Evaluation Criteria in Solid Tumors (RECIST) criteria version 1.1. Survival outcomes were also considered. A total of 220 patients were evaluable for ORR and progression free survival (PFS), whereas all 243 patients were evaluable for overall survival (OS). The median age of the study population was 56 years, and, in terms of stage, 223 patients (92%) presented a metastatic disease, with the remaining 20 (8%) presenting with locally advanced, unresectable disease. Unfortunately, data about patients’ tumor grade are lacking. Univariate and multivariable Cox regression analyses for OS suggested that age > 55 vs. age ≤ 55 years correlated with a worse prognosis (*p* = 0.018 and *p* = 0.01). In the same study, in the Cox regression model for PFS, carried out on 220 evaluable cases, age was not statistically significant. Another retrospective study included 28 advanced Pan-NETs, treated with STZ/5-FU between 2002 and 2018 [20]. The data about tumor grade were available in 25 patients, 5 of them NET G1, 19 G2, and 1 G3 (grade missing in 3 cases). As regards the tumor stage, 26 (92.8%) were metastatic, 22 cases (79%) presented synchronous liver metastases (LM), and 4 metachronous LM. In two cases, the data on tumor stage were lacking. In this study, the median age was 63 years and the analysis of patients’ outcome revealed no significant difference in PFS according to patients’ age > or ≤65 years. Finally, in a retrospective study performed on 84 patients with locally advanced or metastatic pancreatic endocrine carcinoma (islet cell carcinoma) according to 2000/2004 WHO classification (G1 and G2 NET in the latter WHO 2010 classification) treated with the FAS regimen [21], the 2-year PFS was significantly different, i.e., 26% for patients <54 years and 51% for patients ≥ 54 years (*p* = 0.04), whereas the 2-year OS showed no significant difference (65% vs. 76%). Notably, age showed a significant impact on PFS both at univariate and at multivariable analysis (*p* = 0.03 and *p* = 0.005, respectively), with an age lower than median value (equal to 54 years) being associated with worse survival.

#### 3.1.2. Performance Status

The role of performance status (PS) has been evaluated only in one study. In detail, the study by Lahner included 50 well-differentiated Pan-NETs (classified according to 2017 WHO classification) who received the combination STZ/5-FU. The study population consisted of 3 G1, 44 G2 (88%), and 3 G3. A total of 48 patients (96%) presented at least one distant metastatic site, while the remaining 2 cases had only lymph node involvement. The majority of tumors were non-functioning (*n* = 41, 82%) [22]. Eastern Cooperative Oncology Group (ECOG) PS was 0 in 19 cases (38%), 1 in 24 (48%), and 2 in 7 patients (14%). ORR, evaluated according to radiological RECIST 1.1 criteria, was observed in 19 cases: 1 complete response (CR) and 18 partial responses (PRs), accounting for 38% of the population. In 38% of cases, the response was stable disease (SD), and progressive disease (PD) was observed in 24%. ECOG PS did not predict ORR (*p* = 0.695). Among the 43 patients with PS ≤ 1, 17 cases had at least PR (39.5%), 16 SD (37.2%), and 10 PD (23.3%); of the 7 patients with PS = 2, 2 were responders (28.6%), 3 SD (42.9%), and 2 PD (28.6%).

#### 3.1.3. Blood Markers

Four studies have investigated the impact of serum biomarkers on patients’ response to STZ therapy. In a first work, a CgA decrease of more than 30% was associated with a significantly improved ORR (69% vs. 23%; *p* = 0.004) [22]. Rogers found CgA (elevated vs. normal) not to be associated with PFS and OS at multivariate analyses (*p* = 0.20 and *p* = 0.29, respectively) [19]. Another retrospective study included 96 Pan-NETs who received the combination STZ/5-FU between 1998 and 2014 [23]. Tumor grade (classified according to 2010 WHO) was G1 in 11 patients (11.5%), G2 in 76 (79.2%), G3 in 6 (6.3%), and missing in 3 cases (3.1%); tumor stage was III in 6 patients (6.3%) and IV in 90 patients (93.8%). In this study, a reduction of CgA > 30% (observed in 28 cases), compared to a reduction < 30%, correlated (*p* = 0.001) with treatment response measured according to RECIST criteria (version 1.0). Statistical significance was not achieved at univariate and multivariate analysis for time to progression (TTP) and OS (for TTP, *p* = 0.909 and *p* = 0.651; for OS, *p* = 0.117 and *p* = 0.741). In line with this finding, in the study by Kouvaraki, a decrease of CgA > 30% correlated to the response to treatment (ORR) (*p* = 0.04) [21], whereas pre-treatment CgA values, normal vs. increased (available for 60 patients) had no significant impact on 2-years PFS and OS.

#### 3.1.4. Associated Genetic Syndromes

We found a single study evaluating the possible role of genetic syndromes in predicting the response to STZ, namely, the study by Antonodimitrakis [24] performed on 133 Pan-NENs (2010 WHO, G1 = 50, G2 = 48, G3 = 8, unknown = 27; stage I = 2; II = 4; III = 10; IV = 117) treated with a combination of STZ/5-FU in the years 1981–2014. In this retrospective study, the presence of a condition of multiple endocrine neoplasia type 1 (MEN1) did not significantly modulate any of the outcomes evaluated, namely, radiological response, OS, and PFS.

### 3.2. Tumor-Related Features

#### 3.2.1. Tumor Grade

Tumor grade has been evaluated as a predictor of survival in three studies, all with a retrospective design. In a series of 20 patients, all with unresectable or metastatic Pan-NENs (2017 WHO, NET G1 = 3; NET G2 = 13; NET G3 = 3; NEC = 1; stage III = 2, IV = 18) who underwent weekly STZ and oral S1 fluoropyrimidine derivate combined therapy for at least 2 months, Ono found that PFS was not significantly different in NET-G3/NEC-G3 patients compared with NET-G1/G2 patients (*p* = 0.4126) [25]. In another series, the presence of a G3 tumor had a negative impact on survival from the start of treatment at both univariate (*p* < 0.001) and multivariate analyses (*p* = 0.002) [24]. Also, G3 tumors had a significantly shorter PFS (6 months) than G2 (13 months) and G1 (31 months). In the study by Kouravaki, out of 30 patients for whom histological grade information was available, high-grade tumors correlated with shorter PFS (*p* < 0.003 by log-rank test), while the OS did not significantly differ between patients with low- and high-grade tumors [21].

#### 3.2.2. Ki-67 Index

Ki67, together with cell morphology, plays a pivotal role in defining the tumor grade in all gastroenteropancreatic (GEP)-NENs. Five articles evaluated the role of Ki-67 in predicting response to STZ in patients affected by Pan-NEN. In the work by Lahner, comparison of Ki 67 values ≤ 15% (*n* = 32) vs. >15% (*n* = 18) was not associated with a significant impact on patients’ ORR (*p* = 0.99), with rates of 37.5% vs. 38.9% in the two groups, respectively [22]. Ono did not demonstrate a difference in PFS according to the Ki-67 cut-offs employed (<5% vs. >5% *p* = 0.765; <10% vs. > 10%, *p* = 0.322; <15% vs. >15%; *p* = 0.423) [25]. Another study demonstrated only a trend (without a statistically significant difference) on PFS in patients with Ki-67 index ≤ 10% (32 vs. 16 months; *p* = 0.070) [20]. One more study enrolled 110 Pan-NENs, classified according to 2017 WHO as NET G1 in 10 cases, NET G2 in 87, NET G3 in 11, and NEC in 2 cases, with the majority of the patients presenting with stage IV disease (*n* = 109) and the remaining (1 patient) with stage III. Patients received STZ-based chemotherapies schemes, namely, STZ monotherapy (*n* = 90); STZ/5-FU-S1 (*n* = 19) and STZ/DOX (*n* = 1), and a better ORR in the subgroup of patients with Ki-67 index > 5%, (*p* = 0.017) was found [26]. Finally, Dilz reported that the ORR was not different according to Ki-67; however, TTP and OS were significantly shorter in the group of patients with Ki-67 over 15% (at multivariate analysis: *p* = 0.01 and *p* = 0.06, respectively) [23].

#### 3.2.3. Anatomic Primary Tumor Site

A recently published retrospective study performed on 84 patients with unresectable (stage IV) Pan-NENs, treated with STZ/5-FU between 2002 and 2018 (histology available in 28 patients; G1 = 5, G2 = 19, according to 2010 WHO classification; G3 = 4, according 2017 WHO classification) [27], revealed that PR in primary tumors was more frequent among tumors located in the pancreatic tail than those located in the pancreatic head (49% vs. 14%; *p* = 0.03). A trend toward prolonged OS (without reaching statistical significance) was also observed, whereas PFS was not significantly different.

#### 3.2.4. Primary Tumor Size

In the above-mentioned study [27], ORR, PFS, and OS did not significantly differ according to the tumor size (≤50 mm vs. >50 mm). In another retrospective study [25], the tumor size (≤50 mm vs. >50 mm) was not a predictor of response to treatment, based on PFS (*p* = 0.175). 

#### 3.2.5. Tumor Stage

Tumor stage has been evaluated as a predictor of response in three studies. Rogers showed tumor stage not to be a predictor of PFS and OS [19]. In the study by Antonodimitrakis [24], stage IV emerged as a negative predictor factor at multivariate analysis for PFS (*p* < 0.032), whereas the impact of metastatic disease was slightly significant at multivariate analysis for OS (*p* = 0.051). In the study by Kouvaraki, patients with locally advanced tumors (*n* = 2) did not differ from those with metastatic tumors (*n* = 31) in terms of ORR (25% vs. 41%; *p* > 0.05) [21].

#### 3.2.6. Site of Metastasis Origin

In the above-mentioned study by Reher [27], it was also observed that metastases originating from the pancreatic tail achieved a PR to STZ/5-FU more frequently than metastases originating from the pancreatic head (88.5% vs. 41.7%; *p* = 0.005).

#### 3.2.7. Liver Tumor Burden

Severity of liver involvement has been evaluated in three studies. In a multi-center evaluation, Shibuya applied 10, 25, and 50% as cut-off values for liver tumor burden, with no evidence of any trend for radiological response [26]. Similarly, in another study, patients with higher (> 0%) liver tumor burden did not exhibit statistically higher ORR than those with lower (≤10%) involvement (*p* = 0.086, Fisher’s exact test). In the same study, higher liver involvement had no significant impact on TTP and was associated with a statistically significant deterioration of OS at univariate (HR, 2.2; *p* = 0.024) but not at multivariate analysis [23]. In the study by Kouvaraki, minor (defined as ≤75%) liver involvement was not statistically associated with higher ORR, although it was found to be an important prognostic factor related to survival. Indeed, at univariate analysis, the PFS rate at 2 years was 41% (95% CI, 24% to 57%) in the group of patients with LM ≤ 75% (*n* = 61), whereas all the patients with LM of more than 75% (*n* = 12) experienced PD by 14.2 months (*p* < 0.01 by log-rank test). The OS rate at 2 years was 83% for patients with LM ≤ 75%, whereas all patients with LM > 75% died by 15.5 months (*p* < 0.0001). The multivariate survival analysis confirmed that the extent of liver disease was independently associated with shorter PFS and OS [21].

#### 3.2.8. Extrahepatic Spread

The impact of extrahepatic spread has been evaluated in two studies. In the study carried out by Lahner, the involvement of more than two metastatic sites and the presence of bone lesions were not associated with response to treatment (*p* = 0.244 and *p* = 0.237, respectively) [22]. Otherwise, the presence of bone metastases emerged as a negative prognostic factor in terms of OS at univariate and multivariate analyses (*p* = 0.009 and *p* = 0.015, respectively). This impact was not confirmed at univariate and multivariate analysis for PFS (*p* = 0.663 and *p* = 0.711, respectively). In the study by Kouvaraki, the ORR was 19% for the group of patients with extrahepatic metastases with or without liver involvement (*n* = 21), compared with 47% (*p* = 0.03 by Fisher’s exact test) for the group of patients with liver metastases only (*n* = 55) [21].

#### 3.2.9. Functional Status

The potential predictive value of the presence of a hormonal syndrome (functioning Pan-NEN) was evaluated in five studies. A retrospective analysis failed to find a significant difference in ORR between functioning (*n* = 9) and non-functioning (*n* = 41) Pan-NETs (*p* = 0.452) [22]. Another study showed that functional status was a significant factor at univariate Cox regression analysis for OS (*p* = 0.034) but not at multivariate model for OS (*p* = 0.36) and PFS (*p* = 0.15) [19]. In one more study [24] on 100 Pan-NETs evaluable for ORR and PFS, 57 were non-functioning tumors and 43 cases were associated to an hormonal syndrome (gastrinoma in 14 cases, glucagonoma in 8, insulinoma in 6, VIPoma and PTHrp-producing in 3 each, and serotonin-producing in the remaining 2 patients). In terms of PFS, the functional status had a significant impact at univariate (*p* = 0.044) but not at multivariate analysis (*p* = 0.088). Among the 28 cases with a radiological response to the study treatment, 11 had a functioning Pan-NET (1 CR and 10 PR were achieved in these patients). The study by Dilz, including 74 non-functioning and 22 functioning tumors, failed to demonstrate a significant impact of tumor functionality on ORR (*p* = 0.625) [23]. In the study by Kouvaraki, the diagnosis of gastrinoma (*n* = 11) correlated with a statistically significant reduced ORR if compared with other tumor types (*n* = 73), with 0% of response in the case of gastrinomas vs. 45% for the remaining cases (33 responders all among other functioning tumors types together with non-functioning tumors; *p* = 0.002) [21]. The diagnosis of gastrinoma does not have a significant impact on 2-year PFS and 2-year OS, nor on PFS (at both univariate and multivariate analysis). 

#### 3.2.10. Somatostatin Receptors Expression

In the above-mentioned study performed on advanced Pan-NETs [20], no significant difference in PFS for patients with somatostatin receptor imaging (SRI) ^111^Indium-pentetreotide scintigraphy (Octreoscan)-positive (23 patients) vs. negative (5 patients) was found.

#### 3.2.11. Mechanisms of DNA Repair

Only one study evaluated the possible predictive role of O6-methylguanine-methyltransferase (MGMT), a protein involved in the mechanism of DNA damages repairing, in Pan-NEN. In this retrospective study [28], Hijioka aimed at assessing the impact of MGMT deficiency on ORR to STZ, administered in monotherapy or in doublets with 5-FU. The study included 13 patients with advanced well-differentiated Pan-NETs, with a tumor grade of G1 in three cases, G2 in eight, and G3 in twi, according to 2017 WHO classification, who received STZ alone (*n* = 3) or STZ/5-FU (*n* = 10). The study population consisted of 54% of cases with and 46% without MGMT expression (determined by immunohistochemistry). MGMT-negative cases had a significantly higher percentage of PRs, assessed through RECIST criteria version 1.1, as compared with MGMT-positive cases (83.3% vs. 14.3%; *p* = 0.013) [28].

### 3.3. Treatment-Related Factors

#### 3.3.1. Line of Therapy

The line of therapy was evaluated in four studies. The study by Lahner [22] included patients received a combination treatment of STZ and 5-FU as first-line treatment in 27 cases (54%), second-line treatment in 13 (26%), and >second-line in 10 (20%). The impact of STZ/5-FU, evaluated using Fisher’s exact test, as first-line (*n* = 27) vs. >first-line (*n* = 23) therapy was not significant on ORR (*p* = 0.387). The authors pointed out that patients receiving first-line STZ had an OS of 89 months, which dropped to 22 for second-line treatment, and this result was statistically significant for first vs. subsequent therapy lines (*p* = 0.001, log-rank test). However, the authors specify that OS was calculated from the time the drug was administered, this approach not permitting a correct interpretation of the data. Shibuya et al., in their retrospective study, observed no statistically significant difference in ORR between STZ-based chemotherapy as first- or second-line treatment (*p* = 0.490 at univariate analysis and *p* = 0.475 at multivariate model), with ORR of 27.3% for first-line vs. 20.5% for second-line [26]. In the study performed by Dilz, 54 Pan-NETs were treatment-naive (56.3%) and received STZ/5-FU as first-line treatment. Otherwise, 42 of the included patients had received a previous systemic treatment at the time of study start, with the majority of them (*n* = 30) receiving SSAs, 6 received other kinds of chemotherapy, and the remaining 6 patients other no specified treatments. The impact of STZ-5-FU as first-line vs. >first-line was not statistically significant (*p* = 0.413). In addition, the univariate and multivariate analysis confirmed that the treatment line had no significant impact on TTP (*p* = 0.706 and *p* = 0.878, respectively) [23]. In another study, the univariate analysis revealed that patients who received FAS as a second-line chemotherapy showed a statistical trend toward a worse 2-years PFS rate compared with those patients who had not received previous chemotherapy for their disease (*p* = 0.08 by log-rank test), but OS did not significantly differ. Interestingly, multivariate analysis using the Cox proportional hazards model revealed that prior chemotherapy was independently associated with shorter PFS (*p* = 0.01) [21]. 

#### 3.3.2. Response to Prior Treatments

The influence of previous treatments on the efficacy of STZ is another potentially relevant issue to be considered. In a study on 45 patients with advanced well-differentiated pancreatic endocrine carcinoma [29], the authors found that treatment with STZ and DOX, following a previous course of chemotherapy, had a negative prognostic effect on both OR (*p* = 0.0033) and OS (*p* = 0.008). Moreover, they showed a further negative effect of STZ on OS in patients undergoing chemoembolization (*p* = 0.005). This study, however, has some weaknesses, as only 11 of the 45 patients had received previous chemotherapy, and 4 embolization.

**Figure 1:**
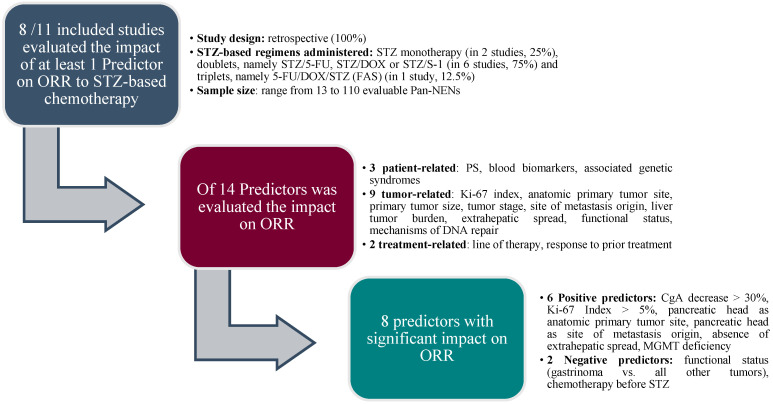
Predictors of response (ORR) to STZ in Pan-NENs. Abbreviations: CgA, Chromogranin A; DOX, doxorubicin; ORR, objective response rate; Pan-NENs, pancreatic neuroendocrine neoplasms; PS, performance status; STZ, Streptozotocin; 5-FU, 5-fluorouracil.

As a final point, given the fundamental role of ORR as an early and accurate indicator of response to treatment, we outlined (Figure 1) the criteria employed to select the eight predictors with significant impact on this key endpoint.

## 4. Discussion

Our study identifies several potential predictors of response to STZ with at least one end-point with statistical significance: age, CgA blood levels, tumor grade, Ki-67% index, anatomical location of the primary tumor, tumor stage, site of metastasis origin, liver tumor burden, extrahepatic spread, functional status, MGMT status, line of therapy, and response to previous treatments.

OS and PFS have been evaluated as outcome measures in the majority of data sources, while the correlation with radiological response has been investigated less extensively. Moreover, for some of the predictors, the findings are conflicting, possibly reflecting heterogeneity in the design of the clinical studies.

For Pan-NENs, age is considered one of the most relevant and well-known prognostic factors. It has been validated by several studies, including patients with either localized or advanced disease. Specifically, higher age has been demonstrated to correlate with reduced OS [30,31,32]. Interestingly, the age cut-off value differs among studies, being 60 years in many works [30,31], and 65 [32,33] or 75 years [34] in others. In line with the literature data, the study by Rogers confirmed a significant impact of higher age (>55 years) on OS [19]. Otherwise, in the study by Kouvaraki [21], age had a not statistically significant impact on OS, albeit a trend for better OS for patients with higher age than the median value was observed. In both cases, the regimen was FAS, but a relevant difference in terms of sample size (243 for Rogers’ study vs. 84 for Kouvaraki’s one) should be considered, suggesting a different statistical power for the two studies. Regarding the impact of age on PFS, literature evidence is also conflicting [35,36] but, overall, shows a worse PFS in older patients. Two of the works included in our analysis did not find a statistically significant effect of age on PFS [19,20], whereas the study by Kouvaraki demonstrated that patients with age lower than the median value (equal to 54 years) had a worse PFS [21]. In this latter case, however, the limited sample size should be taken into account.

CgA is a protein commonly secreted by NENs, including Pan-NENs. CgA is a clinically useful biomarker of NENs with a sensitivity of 66%, specificity of 95%, and an overall accuracy of 71% in Pan-NENs [37,38]. However, it is important to note that CgA levels can be influenced by various factors, including concomitant medical conditions and medications. CgA levels have been linked to tumor burden in Pan-NENs [39,40], whereas its prognostic role is more conflicting. Interestingly, CgA can serve as a biomarker for the evaluation of the therapeutic response in GEP-NETs [41]. Overall, the studies included in our analysis demonstrated that a decrease in CgA levels was associated with an improved ORR. These data are also supported by another retrospective study that included 133 well-differentiated Pan-NETs (2010 WHO classification), treated with the combination of STZ and 5-FU [24]. In this study, 28 of the 100 cases that were radiologically evaluable for the assessment of the response to treatment displayed an objective response, and, specifically, 3 patients had CR and 25 patients PR. Of these 28 Pan-NETs, in 18 cases (64%), a biochemical response with reduced levels of CgA by > 50% was also observed.

Tumor grade, which is determined using measures of tumor proliferation (mitotic index and Ki-67), is often used as a surrogate for the biological aggressiveness of NENs. Indeed, increasing tumor grade correlates with a decrease in OS and PFS in Pan-NEN [42]. The results of our search show that such a relation is substantially valid also for STZ-treated patients. Indeed, the only study reporting the PFS results not to be related with tumor grade is hampered by the exiguous number of participants with NET/NEC G3 [25]. By contrast, the role of tumor grade in determining radiological response to STZ is yet to be investigated.

Ki-67 index is a measure of cellular proliferation, and its role as a prognostic marker in NENs is well-established [4]. Several studies support a different response to chemotherapy in NENs according to Ki-67 value [43]. One of the most relevant, within this context, is represented by the NORDIC study [44], which demonstrated a significant difference in the response to platinum-based chemotherapy according to Ki-67 in advanced gastrointestinal NENs. In this study, patients with Ki-67 < 55% had a lower response rate if compared to cases with Ki-67 > 55% (15% vs. 42%; *p* < 0.001). Further studies have supported this evidence, suggesting a cut-off of Ki-67 equal to 55% to separate patients who mostly benefit from platinum-based chemotherapy, and patients who should be treated preferably with other therapeutic options (as targeted agents or other chemotherapy drugs) [45]. With regard to STZ, a work by Shibuya showed a higher ORR for G2 (23%) compared to G1 (20%) and G3 (18.2%) Pan-NETs. The results of our review cannot establish a definitive role of Ki-67 as a predictive marker in Pan-NENs. However, among the five studies analyzed, two were able to assign to Ki-67 a significant role, namely, a better ORR for Ki-67 > 5% [26], a worse TTP and OS for Ki-67 > 15% [23], and a trend (*p* = 0.070) for a better PFS for Ki-67 < 10% [20]. Otherwise, the remaining two studies failed to demonstrate a significant impact on ORR [22] or on PFS [25]. Moreover, in a retrospective study performed on 77 NENs (mostly pancreatic, *n* = 65, 84.4%) treated with STZ in combination with 5-FU or DOX, the multivariate analysis indicated that PFS was higher in patients with Ki-67 < 10% when compared with patients with Ki-67 ≥ 10% (*p* = 0.034) [46]. Therefore, a possible explanation of these differences in the achieved results and significance across the studies could be found in the Ki-67 cut-off chosen, which is largely heterogeneous in the selected works. We can speculate that, as for Ki-67, a value of 6 to 9% might represent the best target in the evaluation of the response to STZ in Pan-NENs patients. 

The reason for the different response of the primary tumors (and related metastasis) according to the anatomical location of the primary tumor [27] is unclear. Several factors might affect the outcome, such as different site-related genetic profiles and local factors (i.e., tumor microenvironment).

In patients with Pan-NENs, stage is a well-established predictor of prognosis regardless of any other variable [47,48]. However, significant correlations with survival were found only in one of the two studies reporting PFS and OS as outcome measures. In STZ-treated patients, tumor stage also seems not to be of value for radiological response, although such conclusion is supported by a single study.

In patients with Pan-NENs, the liver is the most common site of metastasis, with approximately 28–77% of patients either presenting with synchronous LM or developing metachronous LM in their lifetime [49]. Clinical studies consistently indicate that the occurrence of LM has a detrimental effect on patient prognosis [50,51], and their extension is linked to survival [52,53]. Chemotherapy regimens including STZ are widely recommended in patients with advanced tumors when the tumor burden is high [5,16]. The results of our review show that a lower liver tumor burden is associated with better survival outcomes in Pan-NEN patients treated with STZ-based chemotherapy, irrespective of the cut-off values that are applied to define the extent of involvement [21,23].

The presence of extrahepatic metastases has been demonstrated to have a significant impact on Pan-NEN survival, and that is a crucial point to be considered in the management of these patients [5]. Among the extrahepatic sites, bone secondary lesions are considered a quite rare occurrence in Pan-NEN. However, according to the literature data, about 13% of patients affected by GEP-NET develop bone metastases [54]. Of note, bone metastases have been identified as a negative prognostic factor for PanNEN, although only limited evidence is available. A retrospective monocentric study evaluated 314 Pan-NENs, showing that the survival of patients with bone metastases was significantly reduced when compared with patients without bone lesions (*p* = 0.016) [55]. Another study, including NENs from different primary sites, showed a higher proportion of bone metastases in high-grade NECs than in Pan-NETs (20% vs. 8%) [56]. In this study, the impact of bone metastasis on Pan-NETs survival was not significant, despite a trend suggesting a negative role for this disease localization (*p* = 0.222; OS of 62.1 months for patients with bone metastases vs. 75.4 months for patients without bone lesions). In the study by Lahner, included in our review, a significant impact on patients’ OS was demonstrated for bone metastases, while the significance was not reached in terms of PFS [22]. To date, the role of bone metastases in the therapeutic approach of Pan-NETs has not been clarified [57]. In this context, PRRT has emerged as an effective therapeutic option, also providing an improvement of associated symptoms as bone pain [58,59]. Moreover, the presence of bone lesions has been postulated to be a negative predictor for response to chemotherapy [57], even if there are no conclusive data about this issue.

Focusing on STZ, the studies included in our analysis report conflicting results: one of them showed a not significant impact of more than two metastatic sites as well as of the presence of bone metastases on ORR [22], while the other detected a lower ORR in Pan-NETs with extrahepatic secondary lesions [21]. In the work by Kouvaraki, the site of extrahepatic lesions is not further specified. Therefore, a specific interpretation of the impact of bone lesions on STZ efficacy is not feasible.

The functional status of Pan-NETs, based on hormone secretion, has been postulated to influence the response to STZ. Specifically, non-functioning Pan-NETs are awaited to have a better response to STZ treatment. A potential rationale beyond this difference could be that functioning tumors are often well-differentiated and exhibit slower growth rates, making them less susceptible to the cytotoxic effects of STZ. Few works have evaluated the impact of tumor functionality on response to STZ administered both as single agent and in combination schemes for Pan-NENs; specifically, STZ monotherapy and STZ/5-FU in the study by Moertel [12]; STZ/5-FU or STZ/DOX in the study by Eriksson [60]; STZ/5-FU in the study by Schrader [20]. In these three studies, detailed statistical data are lacking, thus preventing a correct interpretation of the provided data. However, both Eriksson and Schrader found different ORR to STZ-based chemotherapy according to different types of hormonal syndromes (specifically, VIPoma, and insulinoma resulted in increased ORR when compared to other hormonal syndromes). In our analysis, two of the included studies failed to demonstrate a significant impact of tumor functional status on ORR [22,23], in line with available literature data [12,20,60]. Only one work demonstrated a significantly lower ORR in patients with gastrinoma vs. other functioning and non-functioning tumors [21], supporting a differential activity of STZ according to the type of functioning Pan-NET.

MGMT loss has been advocated as a possible positive predictive factor of response for STZ in Pan-NENs [28]. Interesting, in a study performed in NENs of different anatomic sites, including the pancreatic, PFS and OS from first alkylant use (temozolomide, dacarbazine, and STZ) were higher in patients with MGMT protein loss (respectively, 20.2 vs. 7.6 months, *p* < 0.001, and 105 vs. 34 months, *p* = 0.006), thus suggesting that MGMT status is associated with response to alkylant-based chemotherapy in NENs [61]. In a study performed in 2023 by Yagi, of the 19 cases treated with STZ with known MGMT status, 6 cases had SD and 4 cases PD in MGMT-positive patients (*n*  =  10), while 5 cases had PR and 4 SD in MGMT-negative patients (*n*  =  9), and these data support the role of MGMT status in modulating the response to STZ [62]. While the data reported in our review are intriguing, confirmation in prospective controlled studies are expected. The importance of this field is also testified by the high percentage of Pan-NENs that are MGMT-deficient [63,64]. Moreover, additional mechanisms of repair of DNA damage should be explored.

The response to STZ may be influenced by the previous treatments received by the patient. However, there are limited data in the literature on the efficacy of STZ in Pan-NENs in patients who have already received previous therapies, and this is probably attributed to the fact that STZ-based chemotherapy regimens have long been the first line of treatment in patients with NENs. In Delanuoit’s study [29], patients who have not been previously exposed to chemotherapy, or who have received limited prior treatments, have a better response to STZ. In the same direction, the study by Kouvaraki confirmed the line of therapy (specifically, FAS as second-line chemotherapy) as a negative independent prognostic factor for PFS. However, these data were not confirmed in OS [21]. One possible explanation is that patients who have been extensively treated with other chemotherapy agents might have developed resistance mechanisms to other anticancer agents, including STZ. Patients who had received chemoembolization with DOX also showed reduced OS. However, the small number of patients treated with chemoembolization makes the interpretation of data difficult [29]. Finally, a brief observation should be discussed about the role of prior surgery as a potentially impacting factor. In this context, a retrospective study demonstrated, both at univariate as well as at multivariate analysis (*p* = 0.004 and *p* = 0.009, respectively), an increase in OS in a population of 133 patients who had previously undergone surgery [24]. These data were not confirmed at PFS evaluation conducted on 100 of the included patients (univariate *p* = 0.817; multivariate *p* = 0.754). Notably, only 38 of the 133 patients underwent surgery of the primary tumor, and the criteria for choosing surgery are described in detail. Furthermore, this study also included some patients with NENs G3, and the characteristics of the surgically resected patients are not reported [24]. A potential positive prognostic impact of previous surgery was not confirmed by Kouravaki, who, at both univariate and multivariate analyses, found no statistically significant differences in PFS and OS in Pan-NEN patients with previous surgery [21]. In their retrospective study, Rogers et al. also found a non-significant increase in PFS (*p* = 0.57) and OS (*p* = 0.25) in patients with advanced Pan-NENs who received surgery of the primary tumor and were subsequently treated according to the FAS scheme [19]. This was also found in Delanuoit’s study [29]. However, the different results shown in these studies are not easily comparable, both because of the different sample sizes and the different chemotherapy schedules that were used across the various studies. Furthermore, Antonodimitrakis’s study also included NENs G3′s patients; given the high proliferative index, these patients generally respond better to chemotherapy and surgery, ultimately resulting in the reduction of the burden of disease, which could have a positive impact on PFS and OS.

The following features, on the contrary, failed to show any statistically significant end-point: PS, association with genetic syndromes, primary tumor size, and somatostatin receptors expression.

It is well known that patients’ PS can influence treatment response. Generally, patients with good PS and fewer comorbidities tend to have better treatment outcomes in different types of cancer, including NENs [44]. Literature data confirm this observation for NENs treated with chemotherapy [65]; in this study, for 57 NENs (66.7% Pan-NENs) receiving chemotherapy (FOLFOX scheme, an association of 5-FU and oxaliplatin) plus the antiangiogenic bevacizumab, PS of 0 correlated with higher ORR (*p* = 0.034). In the study included in our analysis [22], PS was not found to have a significant impact on response to STZ-based chemotherapy. However, patients with lower PS presented higher ORR and decreased PD rate if compared to cases with PS = 2. Therefore, we cannot rule out that in this case, the limited sample size might have reduced the statistical power to detect significant outcomes.

MEN1 syndrome apparently has no effect on radiological response, OS, and PFS; however, the low number of cases (8 out of 133 subjects, 6%) in the study by Antonodimitrakis [24] might have underscored a possible predictive role.

As for the primary tumor size, no difference in STZ response was found. Coupling these data with the limited capacity to significantly reduce primary Pan-NENs volume, in cases of symptomatic patients with large primary tumors, the benefit of STZ remains to be determined.

Finally, SRI is not correlated with PFS, although this conclusion comes from a single study [20] in which the low number of subjects (*n* = 28) may have affected a possible statistically significant difference. Interestingly, in a work by Krug [46] on 77 NEN patients, mostly Pan-NENs (*n* = 64, 84.4%), a positive Octreoscan (56 out of 70 patients evaluable) indicates that SRI predicts a better ORR (*p* = 0.046). Lastly, we might speculate that newer SRI techniques, with higher sensitivity and specificity, could represent a powerful tool in the prediction of responses.

## 5. Study Limitations

The current study contains some limitations. Most of the evaluated studies did not have the identification of predictors of response to STZ as the primary outcome, and the sample size was therefore not properly calculated for this specific aim. Other limitations are represented by the retrospective nature of many of the selected works, the heterogeneity of the included populations, and thedifferent versions of the WHO classification that has changed across the years. Finally, the chemotherapy regimens employed are also different in the included studies, ranging from STZ administered as monotherapy to the combination of STZ with other anticancer drugs (mainly with the antimetabolite 5-FU, and the anthracycline DOX as doublets, but also in triplets in the FAS scheme).

## 6. Conclusions

In our review, we have detected, summarized, and critically evaluated the possible predictive factors available in the scientific literature with the hope of helping clinicians to maximize the chances of response to STZ in patients with Pan-NENs. Future clinical trials, specifically aimed to elucidate the value of the already-detected factors and eventually identify novel ones, are warranted.

## Figures and Tables

**Table 1:** Characteristics of the selected studies evaluating STZ for pancreatic neuroendocrine neoplasms. These key study data are graphically depicted according to the order in which there are reported in the manuscript.

**(a)** **Patient-related features: age, performance status, blood biomarkers, associated genetic syndromes**								
Predictor	Subgroups compared	End-point evaluated	Predictors’ Significance	*n* evaluable Pan-NENs patients	Treatments	Type of study	First Author	Year
Age	>55 vs. ≤55	OS	Negative	243	5-FU/DOX/STZ (FAS)	retrospective	Rogers JE [19]	2022
Age	>55 vs. ≤55	PFS	Not significant	220	5-FU/DOX/STZ (FAS)	retrospective	Rogers JE [19]	2022
Age	>65 vs. ≤65	PFS	Not significant	28	STZ/5-FU	retrospective	Schrader J [20]	2019
Age	<54 vs. ≥54	2-Year PFS, PFS	Negative	84	5-FU/DOX/STZ (FAS)	retrospective	Kouvaraki MA [21]	2004
Age	<54 vs. ≥54	2-Year OS	Not significant	84	5-FU/DOX/STZ (FAS)	retrospective	Kouvaraki MA [21]	2004
Performance Status	≤1 vs. >1	ORR	Not significant	50	STZ/5-FU	retrospective	Lahner H [22]	2022
Blood biomarkers	CgA decrease > 30%	ORR	Positive	50	STZ/5-FU	retrospective	Lahner H [22]	2022
Blood biomarkers	CgA decrease > 50%	OS	Not significant	243	5-FU/DOX/STZ (FAS)	retrospective	Rogers JE [19]	2022
Blood biomarkers	CgA decrease > 50%	PFS	Not significant	220	5-FU/DOX/STZ (FAS)	retrospective	Rogers JE [19]	2022
Blood biomarkers	CgA decrease > 30%	ORR	Positive	96	STZ/5-FU	retrospective	Dilz DM [23]	2015
Blood biomarkers	CgA decrease > 30%	TTP, OS	Not significant	96	STZ/5-FU	retrospective	Dilz DM [23]	2015
Blood biomarkers	CgA decrease > 30%	ORR	Positive	51	5-FU/DOX/STZ (FAS)	retrospective	Kouvaraki MA [21]	2004
Blood biomarkers	normal vs. increased upon normal values prior to treatment	2-Year PFS, 2-Year OS	Not significant	60	5-FU/DOX/STZ (FAS)	retrospective	Kouvaraki MA [21]	2004
Associated genetic syndromes	Presence vs. absence of MEN1	OS	Not significant	133	STZ/5-FU	retrospective	Antonodimitrakis P [24]	2016
Associated genetic syndromes	Presence vs. absence of MEN1	PFS, ORR	Not significant	100	STZ/5-FU	retrospective	Antonodimitrakis P [24]	2016
Associated genetic syndromes	Presence vs. absence of MEN1	OS	Not significant	100	STZ/5-FU	retrospective	Antonodimitrakis P [24]	2016
**(b) Tumor-related features: tumor grade, Ki-67 index, anatomic primary tumor site** **,** **primary tumor size**								
Predictor	Subgroups compared	End-point evaluated	Predictors’ Significance	*n* evaluable Pan-NENs patients	Treatments	Type of study	First Author	Year
Tumor Grade	NET G1/G2 vs. NET G3/NEC G3	PFS	Not significant	20	STZ/S-1	retrospective	Ono H [25]	2020
Tumor Grade	G3 vs. G1/G2	OS	Negative	133	STZ/5-FU	retrospective	Antonodimitrakis P [24]	2016
Tumor Grade	G3 vs. G1/G2	PFS	Negative	100	STZ/5-FU	retrospective	Antonodimitrakis P [24]	2016
Tumor Grade	Low vs. high	2-Year PFS	Positive	30	5-FU/DOX/STZ (FAS)	retrospective	Kouvaraki MA [21]	2004
Tumor Grade	Low vs. high	2-Year OS	Not significant	30	5-FU/DOX/STZ (FAS)	retrospective	Kouvaraki MA [21]	2004
Ki 67 Index	≤ 15% vs. >15%	ORR	Not significant	50	STZ/5-FU	retrospective	Lahner H [22]	2022
Ki 67 Index	<5 vs. >5%, <10% vs. >10, <15% vs. >15%	PFS	Not significant	20	STZ/S-1	retrospective	Ono H [25]	2020
Ki-67 Index	≤10% vs. >10%	PFS	Not significant	25	STZ/5-FU	retrospective	Schrader J [20]	2019
Ki-67 Index	>5% vs. ≤5%	ORR	Positive	110	STZ monotherapy; STZ/5-FU-S1; STZ/DOX	retrospective	Shibuya H [26]	2018
Ki-67 Index	>15% vs. ≤15%	ORR	Not significant	96	STZ/5-FU	retrospective	Dilz DM [23]	2015
Ki-67 Index	>15% vs. ≤15%	TTP, OS	Negative	96	STZ/5-FU	retrospective	Dilz DM [23]	2015
Anatomic primary tumor site	Head vs. body vs. tail	ORR	Positive	63	STZ/5-FU	retrospective	Reher D [27]	2022
Anatomic primary tumor site	Head vs. body vs. tail	OS	Not significant	72	STZ/5-FU	retrospective	Reher D [27]	2022
Anatomic primary tumor site	Head vs. body vs. tail	PFS	Not significant	67	STZ/5-FU	retrospective	Reher D [27]	2022
Primary tumor size	≤50 mm vs. > 50 mm	ORR, OS, PFS	Not significant	73	STZ/5-FU	retrospective	Reher D [27]	2022
Primary tumor size	≤50 mm vs. > 50 mm	PFS	Not significant	20	STZ/S-1	retrospective	Ono H [25]	2020
**(** **c) Tumor-related features: tumor stage, site of metastasis origin, liver tumor burden, extrahepatic spread**								
Predictor	Subgroups compared	End-point evaluated	Predictors’ Significance	*n* evaluable Pan-NENs patients	Treatments	Type of study	First Author	Year
Tumour stage	Metastatic vs. locally advanced	OS	Not significant	243	5-FU/DOX/STZ (FAS)	retrospective	Rogers JE [19]	2022
Tumour stage	Metastatic vs. locally advanced	PFS	Not significant	220	5-FU/DOX/STZ (FAS)	retrospective	Rogers JE [19]	2022
Tumour stage	Stage 4 vs. others	OS	Not significant	133	STZ/5-FU	retrospective	Antonodimitrakis P [24]	2016
Tumour stage	Stage 4 vs. others	PFS	Negative	100	STZ/5-FU	retrospective	Antonodimitrakis P [24]	2016
Tumour stage	Locally advanced vs. metastatic	ORR	Not significant	33	5-FU/DOX/STZ (FAS)	retrospective	Kouvaraki MA [21]	2004
Site of metastasis origin	Head vs. body vs. tail	ORR	Positive	Not Specified	STZ/5-FU	retrospective	Reher D [27]	2022
Liver tumour burden	10, 25, and 50% as cut-off values	ORR	Not significant	108	STZ; STZ/5-FU-S1; STZ/DOX	retrospective	Shibuya H [26]	2018
Liver tumour burden	≤10% vs. >10%	ORR	Not significant	84	STZ/5-FU	retrospective	Dilz DM [23]	2015
Liver tumour burden	>10% vs. ≤10%	TTP (univariate and multivariate analysis), OS (multivariate analysis)	Not significant	84	STZ/5-FU	retrospective	Dilz DM [23]	2015
Liver tumour burden	>10% vs. ≤10%	OS (univariate analysis)	Negative	84	STZ/5-FU	retrospective	Dilz DM [23]	2015
Liver tumour burden	≤75% vs. >75%	ORR	Not significant	73	5-FU/DOX/STZ (FAS)	retrospective	Kouvaraki MA [21]	2004
Liver tumour burden	≤75% vs. >75%	2-years PFS, 2-years OS	Positive	73	5-FU/DOX/STZ (FAS)	retrospective	Kouvaraki MA [21]	2004
Extrahepatic spread	≥2 distant metastatic sites vs. <2	ORR	Not significant	50	STZ/5-FU	retrospective	Lahner H [22]	2022
Extrahepatic spread	Presence of bone metastases	ORR	Not significant	50	STZ/5-FU	retrospective	Lahner H [22]	2022
Extrahepatic spread	Presence of bone metastases	OS	Negative	50	STZ/5-FU	retrospective	Lahner H [22]	2022
Extrahepatic spread	Presence of bone metastases	PFS	Not significant	50	STZ/5-FU	retrospective	Lahner H [22]	2022
Extrahepatic spread	Liver only vs. other sites ± liver	ORR	Positive	76	5-FU/DOX/STZ (FAS)	retrospective	Kouvaraki MA [21]	2004
**(d) Tumor-related features: functional status, somatostatin receptors expression, mechanisms of DNA repair**								
Predictor	Subgroups compared	End-point evaluated	Predictors’ Significance	*n* evaluable Pan-NENs patients	Treatments	Type of study	First Author	Year
Functional status	Functioning vs. NF	ORR	Not significant	50	STZ/5-FU	retrospective	Lahner H [22]	2022
Functional status	Functioning vs. NF	OS (univariate analysis)	Positive	243	5-FU/DOX/STZ (FAS)	retrospective	Rogers JE [19]	2022
Functional status	Functioning vs. NF	OS (multivariate analysis], PFS	Not significant	243	5-FU/DOX/STZ (FAS)	retrospective	Rogers JE [19]	2022
Functional status	Functioning vs. NF	PFS (univariate analysis)	Positive	100	STZ/5-FU	retrospective	Antonodimitrakis P [24]	2016
Functional status	Functioning vs. NF	PFS (multivariate analysis)	Not significant	100	STZ/5-FU	retrospective	Antonodimitrakis P [24]	2016
Functional status	Functioning vs. NF	ORR	Not significant	96	STZ/5-FU	retrospective	Dilz DM [23]	2015
Functional status	Gastrinoma vs. all other PEC	ORR	Negative	84	5-FU/DOX/STZ (FAS)	retrospective	Kouvaraki MA [21]	2004
Functional status	Gastrinoma vs. all other PEC	2-years PFS, 2-years OS, PFS	Not significant	84	5-FU/DOX/STZ (FAS)	retrospective	Kouvaraki MA [21]	2004
Somatostatin receptors expression	Positive vs. negative Octreoscan	PFS	Not significant	28	STZ/5-FU	retrospective	Schrader J [20]	2019
Mechanisms of DNA repair	MGMT deficiency vs. non-deficiency	ORR	Positive	13	STZ; STZ/5-FU	retrospective	Hijioka S [28]	2019
**(e) Treatment-related factors: line of therapy and response to prior treatment**								
Predictor	Subgroups compared	End-point evaluated	Predictors’ Significance	*n* evaluable Pan-NENs patients	Treatments	Type of study	First Author	Year
Line of therapy	First vs. > first line	ORR	Not significant	50	STZ/5-FU	retrospective	Lahner H [22]	2022
Line of therapy	First vs. > first line	OS	Positive	50	STZ/5-FU	retrospective	Lahner H [22]	2022
Line of therapy	First vs. second line	ORR	Not significant	110	STZ; STZ/5-FU-S1; STZ/DOX	retrospective	Shibuya H [26]	2018
Line of therapy	First vs. second line	2-Year PFS, OS	Not significant	84	5-FU/DOX/STZ (FAS)	retrospective	Kouvaraki MA [21]	2004
Line of therapy	First vs. second line	PFS	Positive	84	5-FU/DOXSTZ (FAS)	retrospective	Kouvaraki MA [21]	2004
Response to prior treatment	Chemotherapy before STZ: yes vs. not	ORR	Negative	45	STZ/DOX	retrospective	Delaunoit T [29]	2004
Response to prior treatment	Chemotherapy before STZ: yes vs. not	OS	Negative	45	STZ/DOX	retrospective	Delaunoit T [29]	2004
Response to prior treatment	Chemoembolization before STZ: yes vs. not	OS	Negative	45	STZ/DOX	retrospective	Delaunoit T [29]	2004

Abbreviations: CgA, Chromogranin A; DOX, doxorubicin; NEC, neuroendocrine carcinoma; NET, neuroendocrine tumor; NF, not functioning; MEN1, multiple endocrine neoplasia type 1; ORR, objective response rate; OS, overall survival; PEC, pancreatic endocrine carcinoma; PFS, progression free survival; STZ, streptozotocin; TTP, time to progression; 5-FU, 5-fluorouracil.

## Data Availability

No new data were created or analyzed in this study. Data sharing is not applicable to this article.

## Supplementary Materials

The following supporting information can be downloaded at https://www.mdpi.com/article/10.3390/jcm12247557/s1: Table S1: Streptozotocin recommendations according to key guidelines for pancreatic neuroendocrine neoplasms, Table S2: WHO classifications of pancreatic neuroendocrine neoplasms from 1980 to 2022 editions.
