# Supplementary material for: Predictive Factors of Response to Streptozotocin in Neuroendocrine Pancreatic Neoplasms
Source: J Clin Med. 2023 Dec 7;12(24):7557. doi: 10.3390/jcm12247557 (PMC10743702; doi:10.3390/jcm12247557)
Supplement: Supplementary file 1 [file jcm-12-07557-s001.zip › jcm-2712738-supplementary.docx]

**Supplementary Table S1.** **Streptozotocin recommendations according to key guidelines for pancreatic neuroendocrine neoplasms**

| Guidelines  Pan-NENs | ENETS | ESMO | JNETS | NCCN |
| --- | --- | --- | --- | --- |
| Advanced G1 Pan-NETs | STZ is recommended in combination with 5-FU or with DOX as one of the therapeutic options after progression to SSAs | STZ is recommended in combination with 5-FU as one of the therapeutic options after progression to SSAs | STZ is recommended as one of the therapeutic options | STZ is recommended as one of the therapeutic options |
| Advanced G2 Pan-NETs | Ki-67 up to 10%: STZ is recommended in combination with 5-FU or with DOX as one of the therapeutic options after progression to SSAs  Ki-67 >10%: STZ is recommended in combination with 5-FU or with DOX as one of the therapeutic options (including first-line therapy) | STZ is recommended in combination with 5-FU as one of the therapeutic options (including first-line therapy) | STZ is recommended as one of the therapeutic options | STZ is recommended as one of the therapeutic options |
| Advanced G3 Pan-NETs | STZ is recommended in combination with 5-FU as one of the therapeutic options (specifically, as first-line therapy) | STZ is recommended in combination with 5-FU as one of the therapeutic options (including first-line therapy) | STZ is recommended as one of the therapeutic options | STZ is recommended as one of the therapeutic options |
| Advanced Pan-NECs | Not recommended | Not recommended | Not recommended | Not recommended |

Abbreviations: DOX, doxorubicin; ENETS, European Neuroendocrine tumor Society; ESMO, European Society for Medical Oncology; NCCN, National Comprehensive Cancer Network; Pan-NECs, pancreatic neuroendocrine carcinomas; Pan-NEN, pancreatic neuroendocrine neoplasms; Pan-NET, pancreatic neuroendocrine tumour; SSA, somatostatin analogue; STZ, streptozotocin; JNETS, Japanese Neuroendocrine Tumor Society; 5-FU, 5-fluorouracil.

**Supplementary Table S2.** **WHO classifications of pancreatic neuroendocrine neoplasms from 1980 to 2022 editions.**

| WHO edition (years) | 1980 | 2000/2004 | 2010 | 2017 | 2022 |
| --- | --- | --- | --- | --- | --- |
| Well-differentiated tumors with low proliferation index | Carcinoid | WDET  Benign; confined to the pancreas; < 2 cm in diameter; <2 mitoses/10 HPF, <2% Ki-67 positive cells;  Uncertain behavior; confined to the pancreas and one or more of the following features: >2 cm in diameter; >2 mitoses/10 HPF; >2% positive cells | NET G1  <2 mitoses/10 HPF, ≤2% Ki-67 | Well-differentiated PanNEN:  PanNET G1  <2 mitoses/10 HPF, <3% Ki-67 | NET G1  <2 mitoses/10 HPF and/or <3% Ki-67 |
| Well-differentiated tumors with intermediate or high proliferation index |  | WDEC  Gross local invasion and/or metastases | NET G2  2–20 mitoses/10 HPF, 3–20% Ki-67 | PanNET G2  2–20 mitoses/10 HPF, 3–20% Ki-67  PanNET G3  >20 mitoses/10 HPF, >20% Ki-67 | NET G2  2–20 mitoses/10 HPF and/or 3–20% Ki-67  NET G3  >20 mitoses/10 HPF and/or >20% Ki-67 |
| Poorly differentiated tumors with high proliferation index |  | PDEC | NEC  >20 mitoses/10 HPF, >20% Ki-67  - Small-cell neuroendocrine carcinoma  - Large-cell neuroendocrine carcinoma | Poorly differentiated PanNEN:  PanNEC  >20 mitoses/10 HPF, >20% Ki-67  - Small-cell type  - Large-cell type | NEC  >20 mitoses/10 HPF and/or >20% Ki-67 (often >70%)  - Small-cell type  - Large-cell type |
| Mixed tumors (neuroendocrine and no neuroendocrine) | - Mucocarcinoid  - Mixed forms carcinoid-adenocarcinomas | Mixed exocrine-endocrine carcinomas | MANEC | MiNEN | MiNEN |

Abbreviations: HPF, high power field; MANEC, mixed adenoneuroendocrine carcinoma; MiNEN, mixed neuroendocrine-non-neuroendocrine neoplasm; NEC, neuroendocrine carcinoma; NET, neuroendocrine tumor; PanNEN, pancreatic neuroendocrine neoplasm; PanNEC, pancreatic neuroendocrine carcinoma; PanNET, pancreatic neuroendocrine tumor; PDEC, poorly differentiated endocrine carcinoma; WDEC, well-differentiated endocrine carcinoma; WDET, well-differentiated endocrine tumor.
